# Supplementary material for: Comparison of radiographic spinal changes and their progression in patients with axial spondyloarthritis vs. psoriatic arthritis with inflammatory axial involvement
Source: Arthritis Res Ther. 2025 Dec 19;28:17. doi: 10.1186/s13075-025-03692-8 (PMC12831346; doi:10.1186/s13075-025-03692-8)
Supplement: Supplementary file 1 — Supplementary Material 1. [file 13075_2025_3692_MOESM1_ESM.docx]

**Supplement**

Figure 1 distribution of time points with mSASSS (method 1) and a-mSASSS (method 2) data


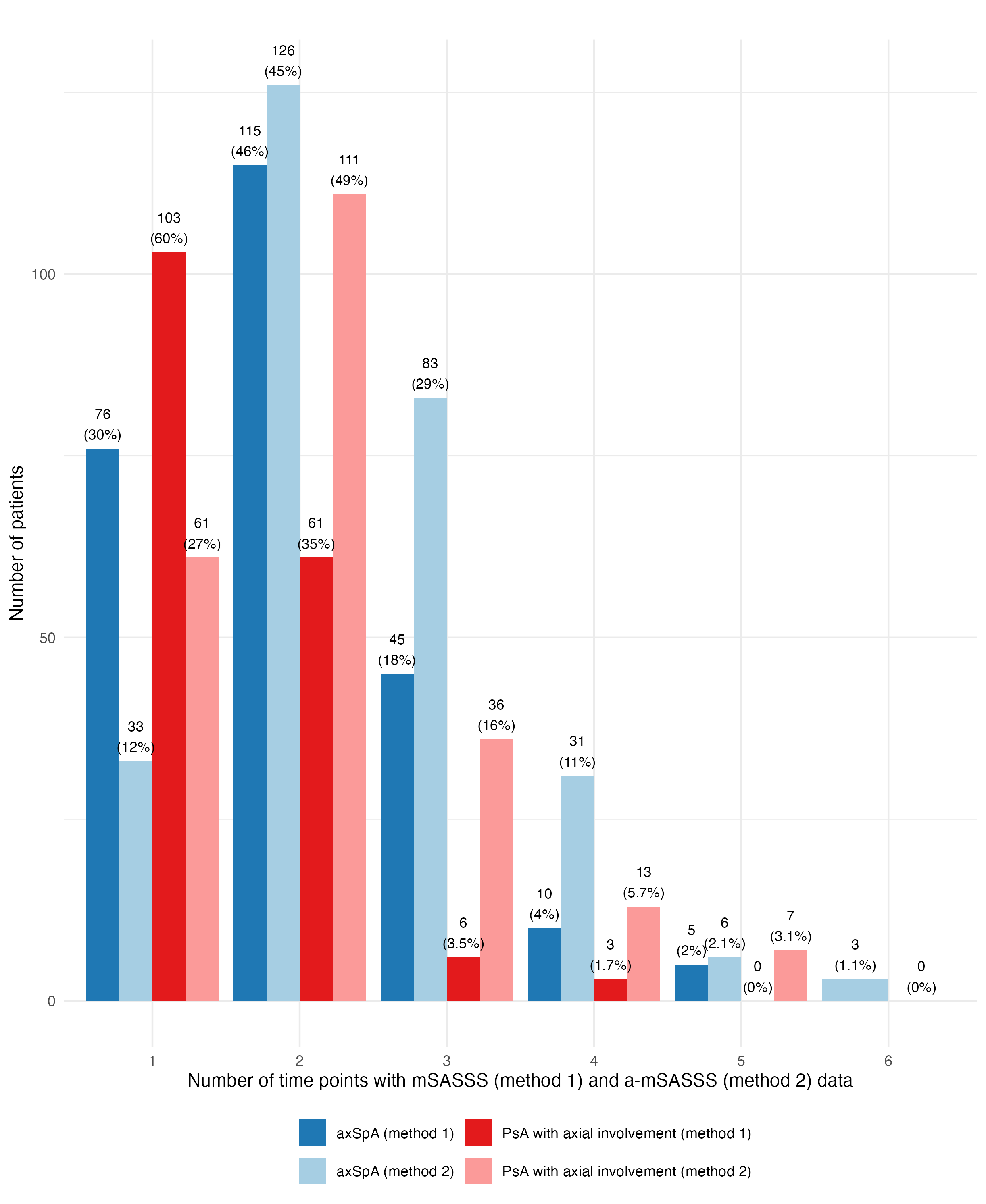


PsA: psoriatic arthritis; axSpA: axial spondyloarthritis; mSASSS: modified Stoke Ankylosing Spondylitis Spinal Score; a-mSASSS: altered modified Stoke Ankylosing Spondylitis Spinal Score calculated independent of the amount of missing vertebral corners
