# Supplementary material for: Comparison of radiographic spinal changes and their progression in patients with axial spondyloarthritis vs. psoriatic arthritis with inflammatory axial involvement
Source: Arthritis Res Ther. 2025 Dec 19;28:17. doi: 10.1186/s13075-025-03692-8 (PMC12831346; doi:10.1186/s13075-025-03692-8)
Supplement: Supplementary file 2 — Supplementary Material 2. [file 13075_2025_3692_MOESM2_ESM.docx]

# Comparison of radiographic spinal changes and their progression in patients with axial spondyloarthritis vs. psoriatic arthritis with inflammatory axial involvement

## Philipp Sewerin^1#^, Imke Redeker^1#^, Eirini Lavda, Gabriela Supp^2^, Daniela Sieghart^2^, Daniel Aletaha^2^, Peter Mandl^2^, David Kiefer^1^, Xenofon Baraliakos^1*^

## # shared first authorship

* Corresponding author; E-Mail: xenofon.baraliakos@elisabethgruppe.de

## ^1^Ruhr-University Bochum, Rheumazentrum Ruhrgebiet, Claudiusstrasse 45, Herne 44649, Germany

^2^Division of Rheumatology, Department of Internal Medicine III, Medical University of Vienna, Vienna, Austria

**Background:** Psoriatic arthritis with inflammatory axial involvement (PsA-ax) and axial spondyloarthritis (axSpA) are distinct entities within the spectrum of spondyloarthritides. Despite overlapping clinical and imaging features, the extent and pattern of radiographic spinal progression in PsA-ax relative to axSpA remain insufficiently characterized.

**Objective:** To describe and analyse differences in radiographic spinal progression between axSpA and PsA-ax.

**Methods:** In this retrospective cohort study, 246 patients (axSpA: n=172; PsA-ax: n=74) with lateral radiographs of the cervical, thoracic, and/or lumbar spine were included. Radiographic progression was quantified using the modified Stoke Ankylosing Spondylitis Spinal Score (mSASSS). An adjusted linear mixed-effects model was used to analyse differences in the longitudinal association with mSASSS between axSpA and PsA-ax. The estimated β-coefficient along with the 95% confidence interval (CI) is reported.

**Results:** The mSASSS differed substantially at baseline, averaging 6 (SD 14) in axSpA and 0.96 (SD 2.12) in PsA-ax. The patient-level mean MSASSS change over 2 years was small overall, but higher in patients with axSpA than in those with PsA-ax, averaging 0.39 (SD 1.83) and 0.07 (SD 0.29), respectively. A total of 27% of axSpA and 5% of PsA-ax had a patient-level mean MSASSS change over 2 years >0 units. In the adjusted linear mixed-effects model, the estimated mean progression in mSASSS over two years was slightly lower in PsA-ax compared to axSpA (adjusted β = -0.088, 95% CI: -0.446 to 0.271).

**Conclusion:** Patients with PsA-ax were found to have slightly lower mean progression in mSASSS over two years than patients with axSpA. However, the wide CI crossing zero indicates large uncertainty and compatibility with no difference in mSASSS progression between PsA with axial involvement and axSpA, warranting further studies.

## Introduction:

Axial spondyloarthritis (axSpA) is a chronic inflammatory disease that affects the axial skeleton^1^. The main symptom is inflammatory back pain caused by inflammation of the sacroiliac joints (sacroiliitis) or spinal structures (spondylitis, facet joint arthritis or enthesitis). The long-term progression of the disease leads to new bone formation in the majority of patients, in particular to the development of syndesmophytes and ankylosis of the costovertebral and costosternal joints^1,2^.

Psoriatic arthritis (PsA) is a systemic inflammatory rheumatic disease associated with bone destruction, joint pain, stiffness, swelling and extra-articular manifestations such as psoriasis, enthesitis and dactylitis^3^. Already Moll and Wright described a predominantly axial manifestation of PsA, which occurs in association with peripheral arthritis. Overall, up to 33% of patients^4^ with PsA have signs for axial inflammation (frequently called PsA with axial involvement), mainly detected as inflammatory back pain. In PsA with axial involvement, typical imaging findings are similar to axSpA, with sacroiliitis, spondylitis and syndesmophyte formation^5^.

Conventional radiographs are still the gold standard for detecting chronic structural changes in the spine, such as erosions or new bone formation, in routine clinical practice^6^. Spinal changes overall and syndesmophytes in particular are reported to be less common in PsA than in axSpA^7^. However, morphologically, the involvement of the spine in PsA patients appears similar to that in axSpA on conventional radiographs, albeit with some important differences: the involvement of the spine is more often unilateral, and the morphology of the syndesmophytes is different, with those in PsA being larger in volume, not exactly following the course of the anterior longitudinal ligament (showing a so-called "paramarginal" localization), and often not occurring in consecutive vertebrae^6,7^. Nevertheless, the distinction between the two entities has become increasingly important recently, especially considering certain therapeutic pathways such as Interleukin-23 inhibition (IL23i), which represent promising treatment targets in PsA^8^, while their potential anti-inflammatory efficacy on the axial skeleton remains unclear^9,10^. Indeed, various clinical studies have demonstrated lack of efficacy of blockade of IL23 or Il12/23 in axSpA^11,12^.

In addition, while radiographic spinal progression in axSpA belongs to the important clinical outcomes, there is substantial lack of information about radiographic damage and progression in patients with inflammatory axial involvement in PsA^13^.

The primary objective of the study was to describe and analyse the radiographic progression of spinal changes in patients with established PsA with axial involvement (PsA-ax) in comparison to axSpA.

## Methods:

In this cohort study, data were retrospectively gathered from two clinical centers (Rheumazentrum Ruhrgebiet, Germany, and Medical University of Vienna, Austria) in 2023. Patients with a primary diagnosis of axSpA and those with PsA-ax that required radiographic examination of the cervical, thoracic and/or lumbar spine in the lateral view were included. Since radiographs were collected based on clinical indication, not all patients had complete sets of images of the entire spine. For the purposes of this study, all available images were taken into account, spanning a period from 1987 to 2023. The available radiographs were evaluated by 2 experienced readers (1 radiologist and 1 rheumatologist), who assigned a score of 0 to 3 to each of the available vertebral corners, based on the radiographic scoring system used in patients with radiographic axSpA (modified Stoke Ankylosing Spondylitis Spine Score (mSASSS^14^). Missing sites were handled as described in the statistical analysis further below.

In addition to the spinal radiographs, demographic data (age, sex), disease specific assessments (Bath Ankylosing Spondylitis Disease Activity Index – BASDAI; Bath Ankylosing Spondylitis Functional Index – BASFI; Disease Activity Score in 28 joints – DAS28 in patients with PsA-ax) and medication (biologic Disease-Modifying Antirheumatic Drugs - bDMARDs), laboratory results (C-reactive protein – CRP; human leukocyte antigen - HLA-B27 status), and lifestyle factors (smoking) were retrieved from electronic health records.

*Exposure*

Diagnosis (axSpA vs PsA-ax) was considered as the exposure.

*Outcome*

Progression in the mSASS score over 2 years was the outcome of interest.

*Covariates*

A directed acyclic graph (DAG) was set up to visualize relationships relevant to the direct effect of diagnosis on mSASSS by utilizing expert knowledge and results from literature. Based on that DAG (figure 1), the adjustment set for the statistical analysis was derived.

**Figure 1 Directed acyclic graph used to estimate the difference in mSASSS progression between axSpA and PsA with axial involvement.**


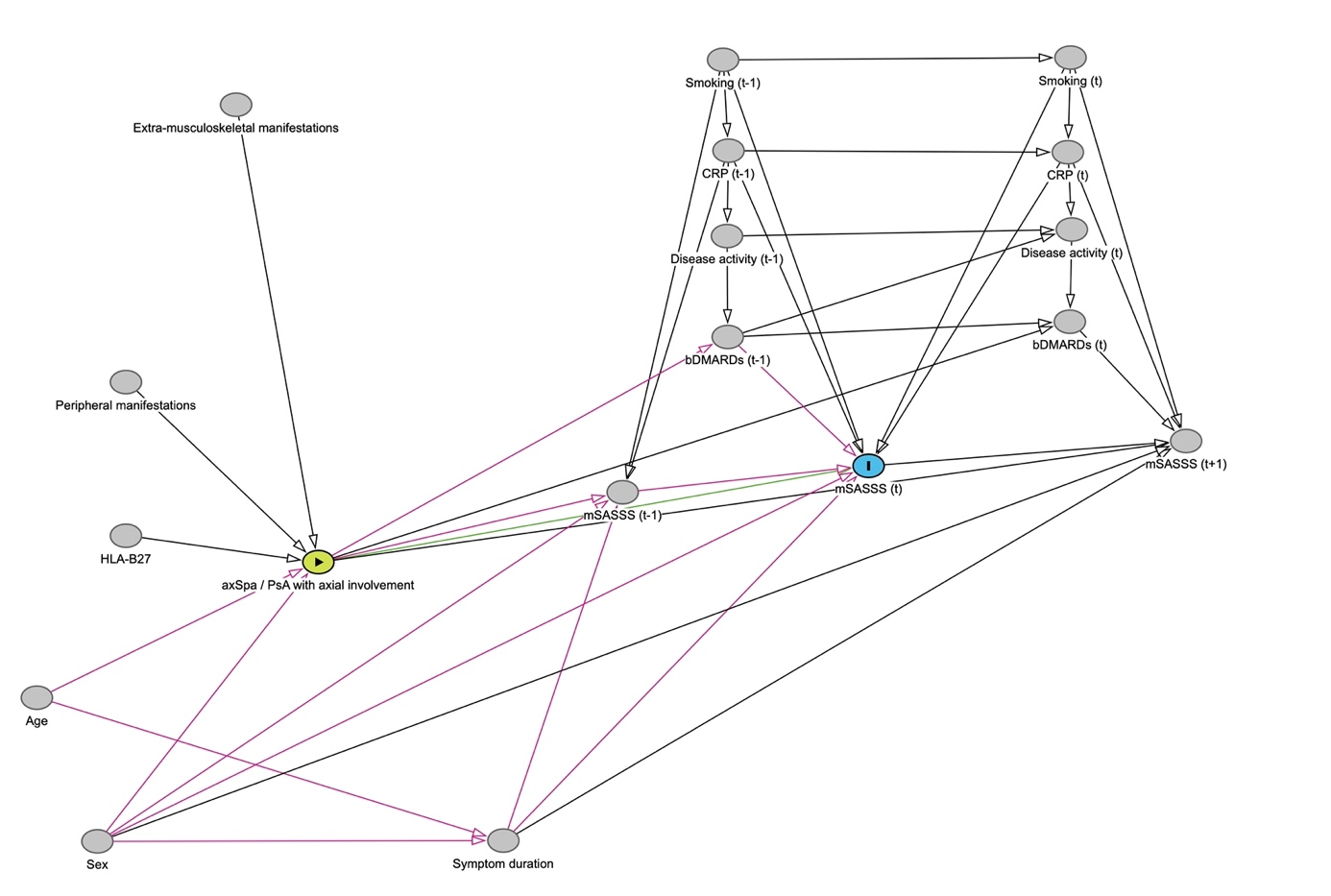


The green node represents the exposure (axSpA vs PsA with axial involvement) and the blue node the outcome (mSASSS) at time point t. The green line represents the direct causal path from the exposure to the outcome, while the pink lines represent biasing paths, which can be corrected for by adjusting for sex, symptom duration, mSASSS at preceding time point, CRP at preceding time point, smoking at preceding time point, bDMARD intake at preceding time point. This DAG is assumed to be representative for every time point t. axSpA: axial spondyloarthritis; bDMARDs: biologic Disease-Modifying Antirheumatic Drugs; CRP: C-reactive protein; HLA-B27, human leucocyte antigen B27; mSASSS: modified Stoke Ankylosing Spondylitis Spine Score; PsA: psoriatic arthritis.

*Statistical analysis*

The mSASS score per reader, patient, and time point was calculated as the sum of the individual vertebral scores as described originally ^14^. If the mSASS score was missing at one time point but non-missing and equal at the previous and subsequent time point, the missing score was imputed with that value. If an individual vertebral score was missing at one time point but had a score of 3 (indicating total ankylosis) at the preceding timepoint, the missing vertebral score was imputed with 3. If an individual vertebral score was missing at one time point but had a score of 0 (indicating no abnormality) at the subsequent timepoint, the missing vertebral score was imputed with 0. The final mSASS score per patient and time point was calculated as the mean mSASS score of both readers. The reliability of the mSASSS assessments between readers was evaluated by the intraclass correlation coefficient.

Descriptive statistics (mean, standard deviation – SD, frequency – n, percentage) were used to describe patient characteristics. Differences between axSpA and PsA-ax in the longitudinal association with mSASSS were analysed using a linear mixed-effects model with the outcome mSASSS as the dependent variable and an interaction term between time (in 2-year intervals) and diagnosis (axSpA vs PsA-ax), adjusted for sex, symptom duration at baseline, mSASSS at preceding visit, CRP at preceding visit, smoking at preceding visit, bDMARD intake at preceding visit and accounting for repeated measurements within patients through a patient-specific random inctercept.

The estimated direct effect (β-coefficient of the interaction term) along with the 95% confidence interval (CI) is reported, reflecting the difference in mSASSS progression between axSpA and PsA-ax.

The DAGs were created using DAGitty, version 3.1, and all statistical analyses were

performed in R, version 4.4.2.

## Results:

**Demographic and clinical characteristics**

Data of 510 patients were retrieved (axSpA = 282; PsA-ax = 228). For the analysis, we focused on the 246 (axSpA = 172; PsA-ax = 74) patients for which data was avialalbe to calculate the mSASSS. The characteristics at baseline of included patients stratified by diagnosis is presented in table 1. Sex distribution differed notably. Patients with axSpA were more often male (60%) than PsA-ax patients, which were more often female (65%). Age was lower in axSpA patients, averaging 46 years (SD 12) compared to 53 years (SD 12) for PsA-ax, while symptom duration was comparable, with mean durations of 10 years (SD 10) for axSpA and 9 years (SD 11) for PsA-ax.

HLA-B27 positivity was considerably higher in axSpA patients (76%) compared to PsA-ax patients (39%) as was bDMARD intake (33% vs 17%), while CRP levels and smoking patterns were comparable in both groups. Disease activity, measured by BASDAI for axSpA and by DAS28 for PsA-ax, was 5.51(SD 2.29) and 4.22 (SD 1.22), respectively.

**Table 1 Patient characteristics at baseline stratified by diagnosis**

|  | **axSpA**, N = 172 | | **PsA** **with axial involvement**, N = 74 | |
| --- | --- | --- | --- | --- |
| **Characteristic** | **n** |  | **n** |  |
| Sex | 172 |  | 74 |  |
| female |  | 69 (40%) |  | 48 (65%) |
| male |  | 103 (60%) |  | 26 (35%) |
| Age (years) | 172 | 46 (12) | 74 | 53 (12) |
| Symptom duration (years) | 154 | 10 (10) | 60 | 9 (11) |
| HLA positive | 147 | 112 (76%) | 28 | 11 (39%) |
| CRP | 102 | 1.62 (3.98) | 35 | 1.60 (3.27) |
| BASDAI | 83 | 5.51 (2.29) | 0 |  |
| BASFI | 74 | 5.54 (2.55) | 0 |  |
| DAS28 | 0 |  | 23 | 4.22 (1.22) |
| bDAMRD therapy | 137 | 46 (34%) | 54 | 9 (17%) |
| Smoking | 152 |  | 60 |  |
| never |  | 61 (40%) |  | 22 (37%) |
| currently |  | 71 (47%) |  | 27 (45%) |
| previously |  | 20 (13%) |  | 11 (18%) |
| mSASSS | 172 | 6 (14) | 74 | 0.96 (2.12) |
| Values are presented as n (%) or mean (SD). PsA: psoriatic arthritis; axSpA: axial spondyloarthritis; BASDAI: Bath Ankylosing Spondylitis Disease Activity Index; BASFI: Bath Ankylosing Spondylitis Functional Index; bDMARD: biologic Disease-Modifying Antirheumatic Drug; CRP: C-reactive protein; DAS28: Disease Activity Score in 28 joints; HLA: Human Leukocyte Antigen; mSASSS: modified Stoke Ankylosing Spondylitis Spine Score. | | | | |


The mSASSS differed substantially at baseline, averaging 6 (SD 14) in axSpA and 0.96 (SD 2.12) in PsA-ax. The distribution of mSASSS at baseline is shown in Figure 2. The intraclass correlation coefficient measuring agreement between readers was 0.86 (95% CI 0.65 to 0.93).

**Figure 2 Distribution of mSASSS at baseline stratified by diagnosis**

**
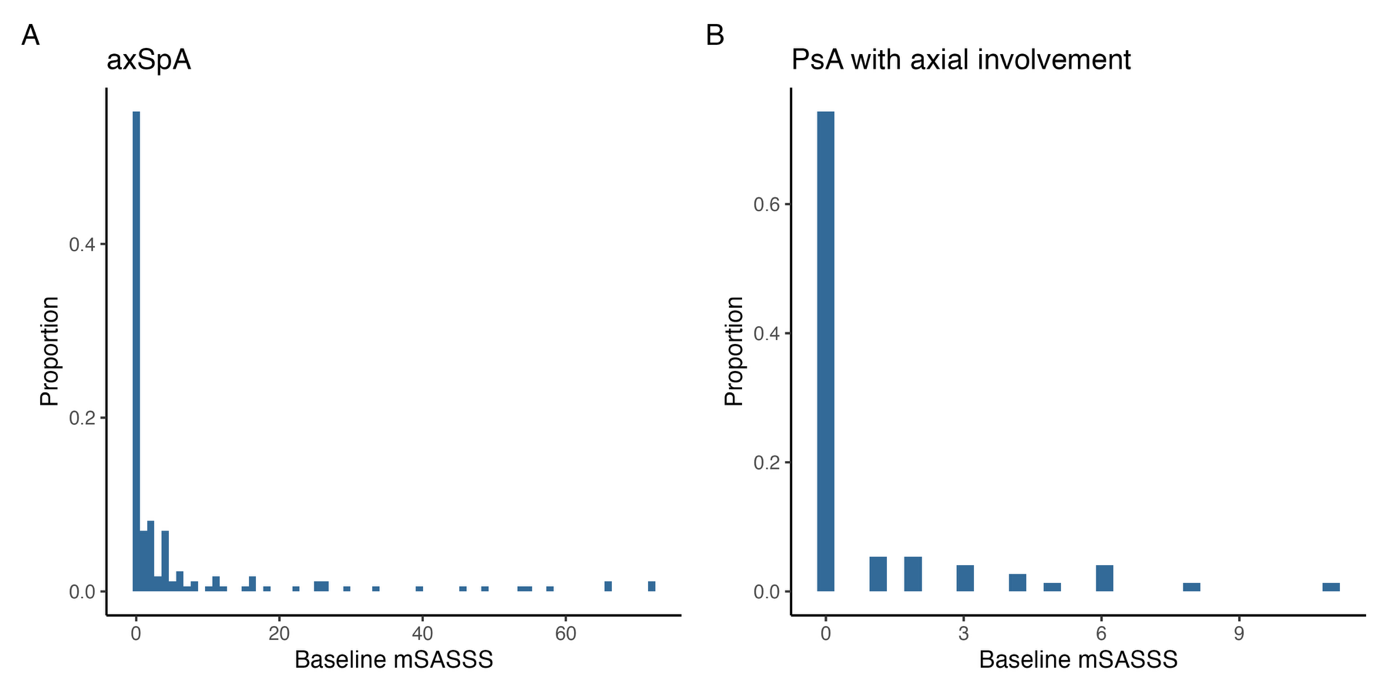
**

axSpA: axial spondyloarthritis, mSASSS: modified Stoke Ankylosing Spondylitis Spine Score; PsA: psoriatic arthritis.

The majority of patients had follow-up images available (table 2) to calculate the change in mSASSS. The mean follow-up time was 5.1 (SD 4.7) years and 2.8 (SD 3.7) years in axSpA and PsA-ax, respectively.

**Table 2 Number of visits stratified by diagnosis**

|  | | **Number of visits** | | | | | | |
| --- | --- | --- | --- | --- | --- | --- | --- | --- |
|  |  | **1** | **2** | **3** | **4** | **5** | **6** | **7** |
| **Diagnosis** | **Axial spondyloarthritis** | 23 | 67 | 43 | 24 | 9 | 4 | 2 |
|  | **Psoriatic arthritis with axial involvement** | 31 | 22 | 14 | 6 | 1 | 0 | 0 |

The patient-level mean MSASSS change over 2 years was higher in patients with axSpA than in those with PsA-ax, averaging 0.39 (SD 1.83) and 0.07 (SD 0.29), respectively. Its distribution is shown in Figure 3. A total of 46 patients (27%) with axSpA and the 4 with PsA-ax (5%) had a patient-level mean MSASSS change over 2 years >0 units, with an average of 1.71 (SD 2.14) units and 0.83 (SD 0.48), respectively.

**Figure 3 Distribution of patient-level mean mSASSS change over 2 years^1^ stratified by diagnosis**

**
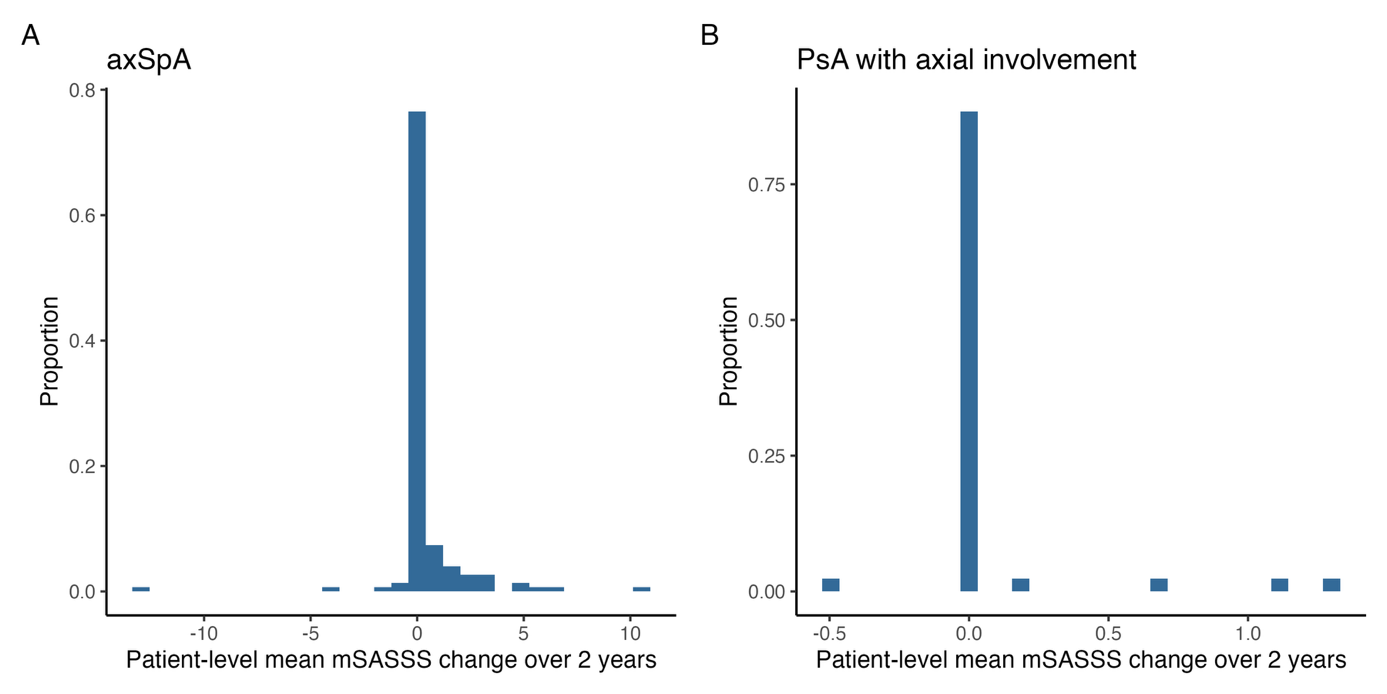
**

^1^ The patient-level mean change in mSASSS over a 2-year period was calculated by linearly interpolating /extrapolating the difference between consecutive measurements, with multiple changes per patient then averaged. axSpA: axial spondyloarthritis, mSASSS: modified Stoke Ankylosing Spondylitis Spine Score; PsA: psoriatic arthritis.

In the adjusted linear mixed-effects model, the estimated mean progression in mSASSS over two years was slightly lower in PsA-ax compared to axSpA (adjusted β = -0.088, 95% CI: -0.446 to 0.271). Moreover, the wide CI crossing zero indicates large uncertainty and compatibility with no difference in mSASSS progression between PsA-ax and axSpA.

## Discussion

Axial skeletal involvement represents a central manifestation in patients with spondyloarthritides. One main goal of therapy is to slow or even stop radiographic progression, which is associated with increasing loss of function and disability^15^. Inflammatory axial involvement has been considered to be important also for patients with PsA. Specific clinical trials for such patients have not been conducted, except for the MAXIMISE study^16^. In general, all available therapies for PsA patients who have documented inflammatory axial involvement and are mentioned in the EULAR or GRAPPA guidelines have mainly been adopted from the treatment of axSpA^17,18^. Overall, one of the most important outcomes remains the effect of these treatments on radiographic progression.

So far, various studies have described similarities and differences between axSpA and PsA-ax , although these have generally been limited to clinical differences. Benavent et al described few clinical differences between the two cohorts except for peripheral involvement, which was significantly more common in the PsA-ax cohort^19^. Regierer et al reported in the Rabbit SpA registry that patients with PsA-ax were older, more frequently female and less frequently HLA B27 positive^13^. The same applies to the cohort we studied, in which we found more women in the PsA-ax group, while axSpA patients were more often HLA B27 positive and younger on average.

To the best of our knowledge, this is the first study investigating differences between axSpA and PsA-ax in the association with mSASSS progression. In this study, axSpA patients showed considerably more radiographic damage at baseline than PsA-ax patients. Although the radiographic progression was low overall, it was estimated in an adjusted analysis to be higher in axSpA than in PsA-ax, albeit with a wide confidence interval, consistent with no difference in radiographic progression between the groups. It should be noted that, in contrast to other studies, only patients with confirmed PsA-ax based on clinical and imaging presentation were included and compared rather than an entire cohort of PsA patients as done in the other studies. Requiring available spinal radiographs may introduce selection bias, particularly affecting the PsA-ax cohort, since routine spinal imaging is less standardized in PsA than in axSpA. Patients with PsA-ax included in this analysis likely represent those with higher clinical suspicion for structural damage, which could have led to over-estimation of radiographic progression in PsA-ax and, thus, likely narrowing the difference in radiographic progression between both groups.

In summary, we describe and analyse differences in radiographic progression between axSpA and PsA-ax patients in this study. This adds to the current debate about possible similarities and differences between both groups. Patients with PsA-ax were found to have slightly lower mean progression in mSASSS over two years than patients with axSpA. However, the wide CI crossing zero indicates large uncertainty and compatibility with no difference in mSASSS progression between PsA with axial involvement and axSpA. Further studies are warranted to reduce uncertainty.

**Declarations**

**Ethics approval and consent to participate**

All patients gave their consent for usage and publication of results based on their  clinical data and images.

**Data availability**

Not applicable

**Competing interests**

The authors declare no competing interests.

**Funding**

Not applicable

**Authors' contributions**

PS, IR, PM and XB designed the study, PS, IR and XB analysed the data, PS, IR, EL, GS, DS, DA, PM, DK and XB interpreted the data, PS, IR and XB wrote the draft, All authors reviewed the manuscript.

**Consent for publication**

Not applicable

**Acknowledgements**

Not applicable

## Literature

1. Robinson, P. C., Van Der Linden, S., Khan, M. A. & Taylor, W. J. Axial spondyloarthritis: concept, construct, classification and implications for therapy. *Nat Rev Rheumatol* **17**, 109–118 (2021).

2. Robinson, P. C., Sengupta, R. & Siebert, S. Non-Radiographic Axial Spondyloarthritis (nr-axSpA): Advances in Classification, Imaging and Therapy. *Rheumatol Ther* **6**, 165–177 (2019).

3. FitzGerald, O. *et al.* Psoriatic arthritis. *Nat Rev Dis Primers* **7**, 59 (2021).

4. Ogdie, A. *et al.* Evaluation of Clinical Diagnosis of Axial Psoriatic Arthritis (PsA) or Elevated Patient-reported Spine Pain in CorEvitas’ PsA/Spondyloarthritis Registry. *The Journal of Rheumatology* **49**, 281–290 (2022).

5. Moll, J. M. H. & Wright, V. Psoriatic arthritis. *Seminars in Arthritis and Rheumatism* **3**, 55–78 (1973).

6. Baraliakos, X. & Braun, J. Imaging Scoring Methods in Axial Spondyloarthritis. *Rheum. Dis. Clin. North Am.* **42**, 663–678 (2016).

7. Baraliakos, X., Coates, L. C. & Braun, J. The involvement of the spine in psoriatic arthritis. *Clinical and Experimental Rheumatology* **33**, S31-5 (2015).

8. Krueger, J. G. *et al.* IL-23 past, present, and future: a roadmap to advancing IL-23 science and therapy. *Front. Immunol.* **15**, 1331217 (2024).

9. Mease, P. J. *et al.* Efficacy of Guselkumab on Axial-Related Symptoms Through up to 2 Years in Adults with Active Psoriatic Arthritis in the Phase 3, Randomized, Placebo-Controlled DISCOVER-2 Study. *Rheumatol Ther* **10**, 1637–1653 (2023).

10. Gladman, D. D. *et al.* Efficacy and safety of guselkumab in biologic-naïve patients with active axial psoriatic arthritis: study protocol for STAR, a phase 4, randomized, double-blinded, placebo-controlled trial. *Trials* **23**, 743 (2022).

11. Baeten, D. *et al.* Risankizumab, an IL-23 inhibitor, for ankylosing spondylitis: results of a randomised, double-blind, placebo-controlled, proof-of-concept, dose-finding phase 2 study. *Ann Rheum Dis* **77**, 1295–1302 (2018).

12. Deodhar, A. *et al.* Three Multicenter, Randomized, Double‐Blind, Placebo‐Controlled Studies Evaluating the Efficacy and Safety of Ustekinumab in Axial Spondyloarthritis. *Arthritis & Rheumatology* **71**, 258–270 (2019).

13. Regierer, A. C. *et al.* Comparison of patients with axial PsA and patients with axSpA and concomitant psoriasis: an analysis of the German register RABBIT-SpA. *RMD Open* **9**, e002837 (2023).

14. Creemers, M. C. W. *et al.* Assessment of outcome in ankylosing spondylitis: an extended radiographic scoring system. *Ann Rheum Dis* **64**, 127–129 (2005).

15. Ramiro, S. *et al.* ASAS-EULAR recommendations for the management of axial spondyloarthritis: 2022 update. *Ann Rheum Dis* **82**, 19 (2023).

16. Baraliakos, X. *et al.* Secukinumab in patients with psoriatic arthritis and axial manifestations: results from the double-blind, randomised, phase 3 MAXIMISE trial. *Ann Rheum Dis* **80**, 582–590 (2021).

17. Gossec, L. *et al.* EULAR recommendations for the management of psoriatic arthritis with pharmacological therapies: 2023 update. *Ann Rheum Dis* ard-2024-225531 (2024) doi:10.1136/ard-2024-225531.

18. Coates, L. C. *et al.* Group for Research and Assessment of Psoriasis and Psoriatic Arthritis (GRAPPA): updated treatment recommendations for psoriatic arthritis 2021. *Nat Rev Rheumatol* **18**, 465–479 (2022).

19. Benavent, D. *et al.* Axial spondyloarthritis and axial psoriatic arthritis: similar or different disease spectrum? *Therapeutic Advances in Musculoskeletal* **12**, 1759720X20971889 (2020).

20. Wanders, A. J. B. *et al.* What is the most appropriate radiologic scoring method for ankylosing spondylitis? A comparison of the available methods based on the Outcome Measures in Rheumatology Clinical Trials filter. *Arthritis Rheum* **50**, 2622–2632 (2004).
